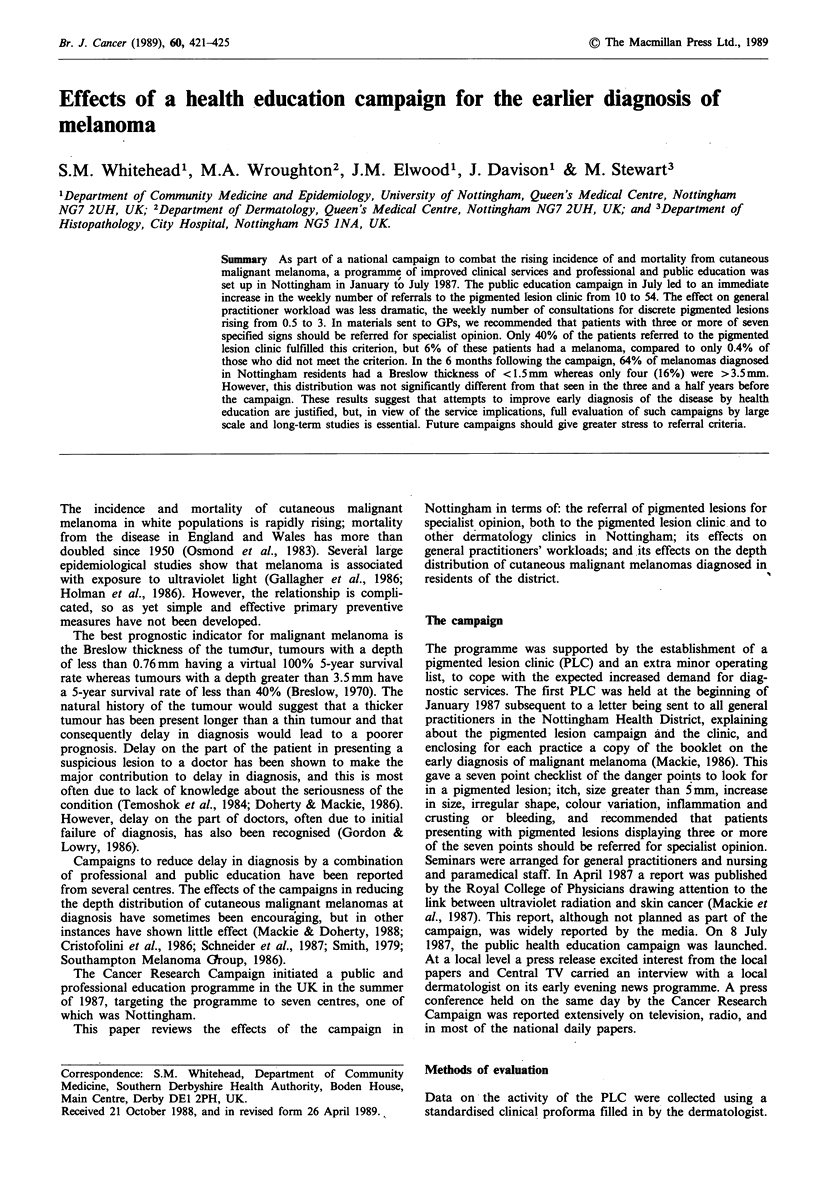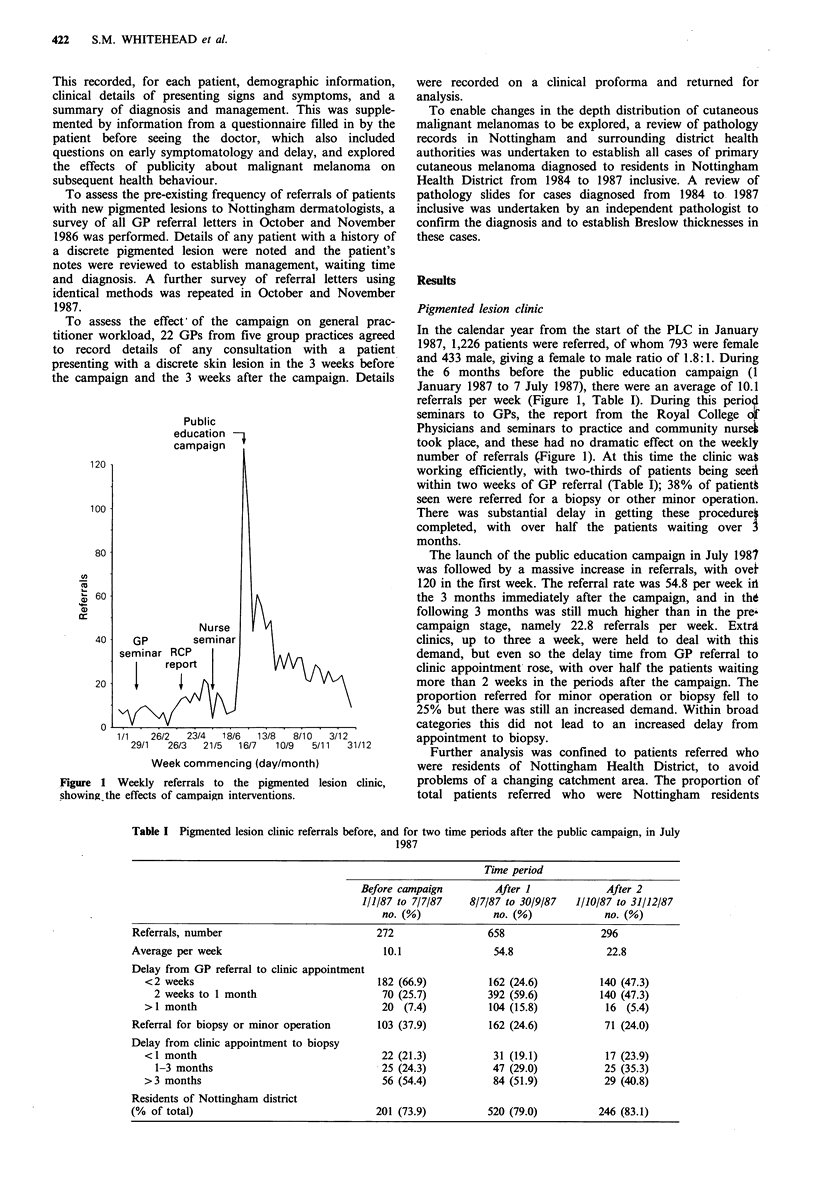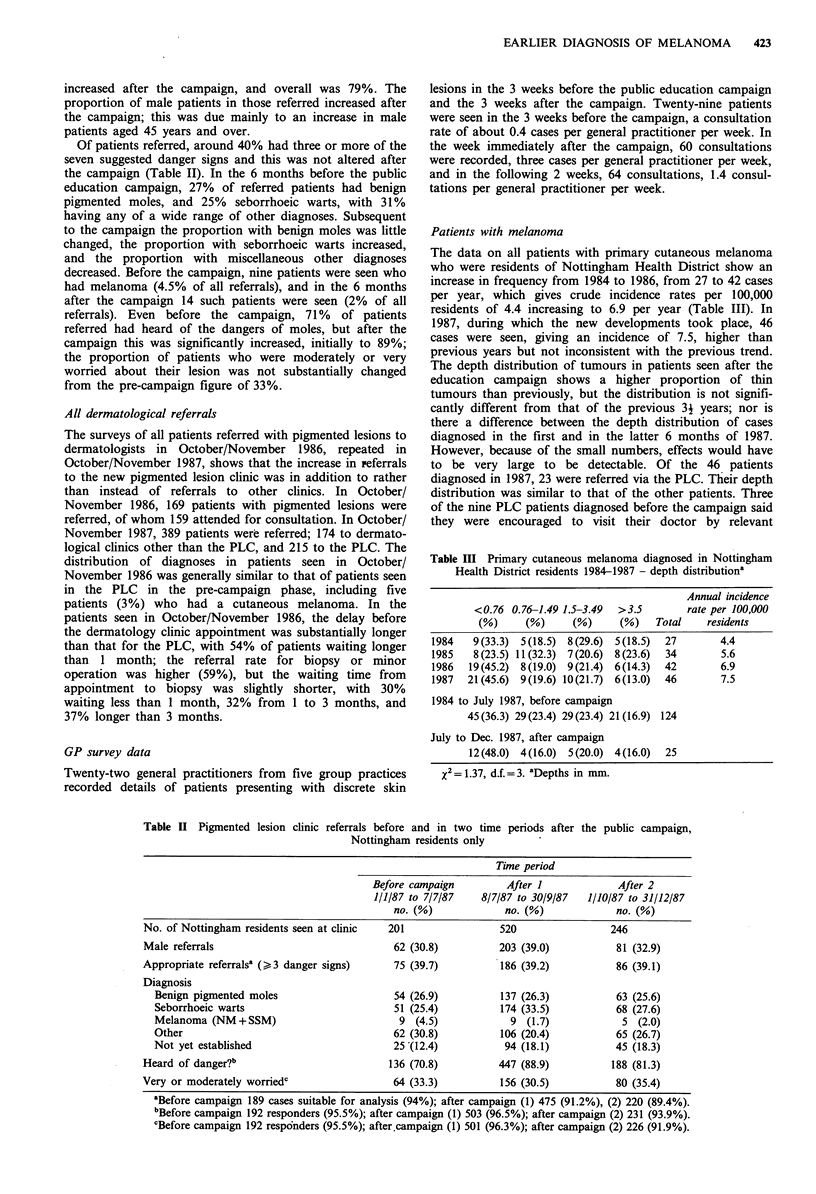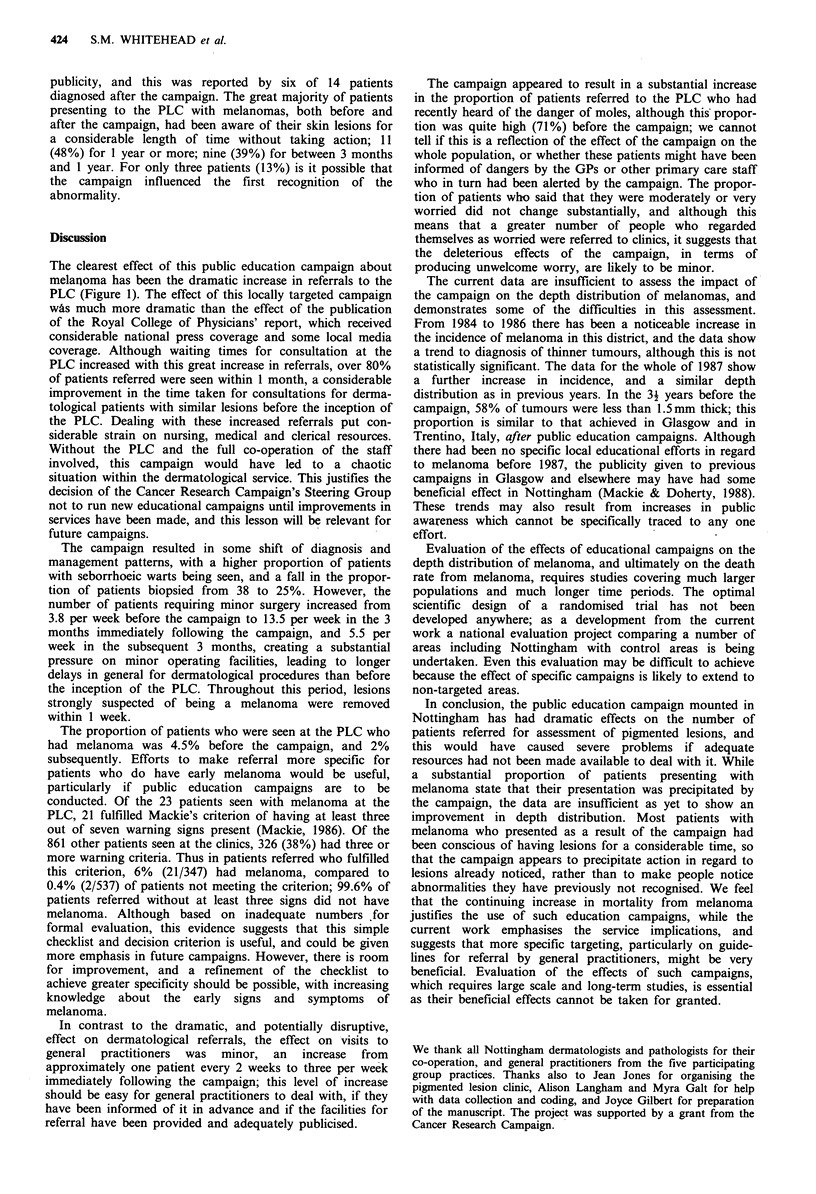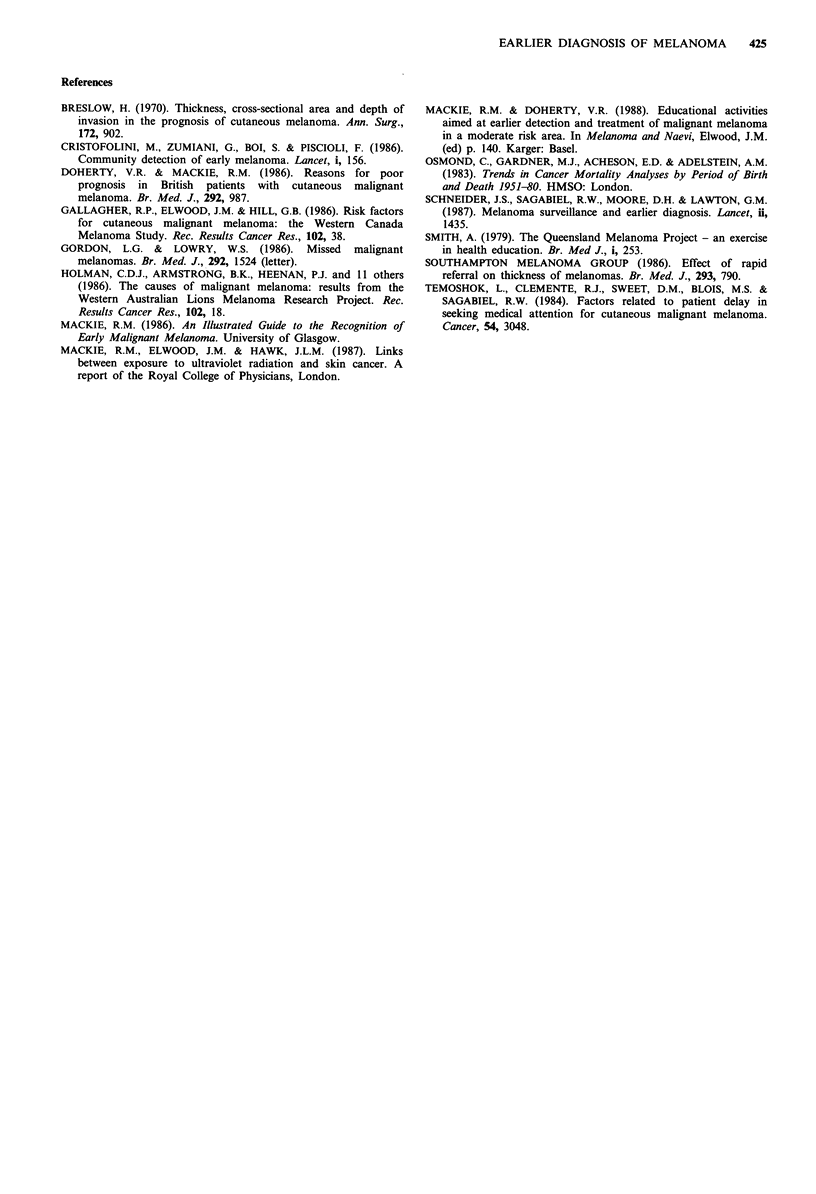# Effects of a health education campaign for the earlier diagnosis of melanoma.

**DOI:** 10.1038/bjc.1989.298

**Published:** 1989-09

**Authors:** S. M. Whitehead, M. A. Wroughton, J. M. Elwood, J. Davison, M. Stewart

**Affiliations:** Department of Community Medicine and Epidemiology, University of Nottingham, Queen's Medical Centre, UK.

## Abstract

As part of a national campaign to combat the rising incidence of and mortality from cutaneous malignant melanoma, a programme of improved clinical services and professional and public education was set up in Nottingham in January to July 1987. The public education campaign in July led to an immediate increase in the weekly number of referrals to the pigmented lesion clinic from 10 to 54. The effect on general practitioner workload was less dramatic, the weekly number of consultations for discrete pigmented lesions rising from 0.5 to 3. In materials sent to GPs, we recommended that patients with three or more of seven specified signs should be referred for specialist opinion. Only 40% of the patients referred to the pigmented lesion clinic fulfilled this criterion, but 6% of these patients had a melanoma, compared to only 0.4% of those who did not meet the criterion. In the 6 months following the campaign, 64% of melanomas diagnosed in Nottingham residents had a Breslow thickness of less than 1.5 mm whereas only four (16%) were greater than 3.5 mm. However, this distribution was not significantly different from that seen in the three and a half years before the campaign. These results suggest that attempts to improve early diagnosis of the disease by health education are justified, but, in view of the service implications, full evaluation of such campaigns by large scale and long-term studies is essential. Future campaigns should give greater stress to referral criteria.


*Br. J. Cancer* (1989), **60**, 421–425 © The Macmillan Press Ltd., 1989

### Effects of a health education campaign for the earlier diagnosis of melanoma

S.M. Whitehead[1], M.A. Wroughton[2], J.M. Elwood[1], J. Davison[1] & M. Stewart[3]

[1]*Department of Community Medicine and Epidemiology, University of Nottingham, Queen's Medical Centre, Nottingham NG7 2UH, UK;* [2]*Department of Dermatology, Queen's Medical Centre, Nottingham NG7 2UH, UK; and* [3]*Department of Histopathology, City Hospital, Nottingham NG5 1NA, UK.*

**Summary** As part of a national campaign to combat the rising incidence of and mortality from cutaneous malignant melanoma, a programme of improved clinical services and professional and public education was set up in Nottingham in January to July 1987. The public education campaign in July led to an immediate increase in the weekly number of referrals to the pigmented lesion clinic from 10 to 54. The effect on general practitioner workload was less dramatic, the weekly number of consultations for discrete pigmented lesions rising from 0.5 to 3. In materials sent to GPs, we recommended that patients with three or more of seven specified signs should be referred for specialist opinion. Only 40% of the patients referred to the pigmented lesion clinic fulfilled this criterion, but 6% of these patients had a melanoma, compared to only 0.4% of those who did not meet the criterion. In the 6 months following the campaign, 64% of melanomas diagnosed in Nottingham residents had a Breslow thickness of <1.5 mm whereas only four (16%) were >3.5 mm. However, this distribution was not significantly different from that seen in the three and a half years before the campaign. These results suggest that attempts to improve early diagnosis of the disease by health education are justified, but, in view of the service implications, full evaluation of such campaigns by large scale and long-term studies is essential. Future campaigns should give greater stress to referral criteria.

The incidence and mortality of cutaneous malignant melanoma in white populations is rapidly rising; mortality from the disease in England and Wales has more than doubled since 1950 (Osmond *et al.*, 1983). Several large epidemiological studies show that melanoma is associated with exposure to ultraviolet light (Gallagher *et al.*, 1986; Holman *et al.*, 1986). However, the relationship is complicated, so as yet simple and effective primary preventive measures have not been developed.

The best prognostic indicator for malignant melanoma is the Breslow thickness of the tumour, tumours with a depth of less than 0.76 mm having a virtual 100% 5-year survival rate whereas tumours with a depth greater than 3.5 mm have a 5-year survival rate of less than 40% (Breslow, 1970). The natural history of the tumour would suggest that a thicker tumour has been present longer than a thin tumour and that consequently delay in diagnosis would lead to a poorer prognosis. Delay on the part of the patient in presenting a suspicious lesion to a doctor has been shown to make the major contribution to delay in diagnosis, and this is most often due to lack of knowledge about the seriousness of the condition (Temoshok *et al.*, 1984; Doherty & Mackie, 1986). However, delay on the part of doctors, often due to initial failure of diagnosis, has also been recognised (Gordon & Lowry, 1986).

Campaigns to reduce delay in diagnosis by a combination of professional and public education have been reported from several centres. The effects of the campaigns in reducing the depth distribution of cutaneous malignant melanomas at diagnosis have sometimes been encouraging, but in other instances have shown little effect (Mackie & Doherty, 1988; Cristofolini *et al.*, 1986; Schneider *et al.*, 1987; Smith, 1979; Southampton Melanoma Group, 1986).

The Cancer Research Campaign initiated a public and professional education programme in the UK in the summer of 1987, targeting the programme to seven centres, one of which was Nottingham.

This paper reviews the effects of the campaign in Nottingham in terms of: the referral of pigmented lesions for specialist opinion, both to the pigmented lesion clinic and to other dermatology clinics in Nottingham; its effects on general practitioners' workloads; and its effects on the depth distribution of cutaneous malignant melanomas diagnosed in residents of the district.

### The campaign

The programme was supported by the establishment of a pigmented lesion clinic (PLC) and an extra minor operating list, to cope with the expected increased demand for diagnostic services. The first PLC was held at the beginning of January 1987 subsequent to a letter being sent to all general practitioners in the Nottingham Health District, explaining about the pigmented lesion campaign and the clinic, and enclosing for each practice a copy of the booklet on the early diagnosis of malignant melanoma (Mackie, 1986). This gave a seven point checklist of the danger points to look for in a pigmented lesion; itch, size greater than 5 mm, increase in size, irregular shape, colour variation, inflammation and crusting or bleeding, and recommended that patients presenting with pigmented lesions displaying three or more of the seven points should be referred for specialist opinion. Seminars were arranged for general practitioners and nursing and paramedical staff. In April 1987 a report was published by the Royal College of Physicians drawing attention to the link between ultraviolet radiation and skin cancer (Mackie *et al.*, 1987). This report, although not planned as part of the campaign, was widely reported by the media. On 8 July 1987, the public health education campaign was launched. At a local level a press release excited interest from the local papers and Central TV carried an interview with a local dermatologist on its early evening news programme. A press conference held on the same day by the Cancer Research Campaign was reported extensively on television, radio, and in most of the national daily papers.

### Methods of evaluation

Data on the activity of the PLC were collected using a standardised clinical proforma filled in by the dermatologist.

Correspondence: S.M. Whitehead, Department of Community Medicine, Southern Derbyshire Health Authority, Boden House, Main Centre, Derby DE1 2PH, UK.
Received 21 October 1988, and in revised form 26 April 1989.

This recorded, for each patient, demographic information, clinical details of presenting signs and symptoms, and a summary of diagnosis and management. This was supplemented by information from a questionnaire filled in by the patient before seeing the doctor, which also included questions on early symptomatology and delay, and explored the effects of publicity about malignant melanoma on subsequent health behaviour.

To assess the pre-existing frequency of referrals of patients with new pigmented lesions to Nottingham dermatologists, a survey of all GP referral letters in October and November 1986 was performed. Details of any patient with a history of a discrete pigmented lesion were noted and the patient's notes were reviewed to establish management, waiting time and diagnosis. A further survey of referral letters using identical methods was repeated in October and November 1987.

To assess the effect of the campaign on general practitioner workload, 22 GPs from five group practices agreed to record details of any consultation with a patient presenting with a discrete skin lesion in the 3 weeks before the campaign and the 3 weeks after the campaign. Details were recorded on a clinical proforma and returned for analysis.

To enable changes in the depth distribution of cutaneous malignant melanomas to be explored, a review of pathology records in Nottingham and surrounding district health authorities was undertaken to establish all cases of primary cutaneous melanoma diagnosed to residents in Nottingham Health District from 1984 to 1987 inclusive. A review of pathology slides for cases diagnosed from 1984 to 1987 inclusive was undertaken by an independent pathologist to confirm the diagnosis and to establish Breslow thicknesses in these cases.

**Figure 1** Weekly referrals to the pigmented lesion clinic, showing the effects of campaign interventions.

### Results

### Pigmented lesion clinic

In the calendar year from the start of the PLC in January 1987, 1,226 patients were referred, of whom 793 were female and 433 male, giving a female to male ratio of 1.8:1. During the 6 months before the public education campaign (1 January 1987 to 7 July 1987), there were an average of 10.1 referrals per week (Figure 1, Table I). During this period seminars to GPs, the report from the Royal College of Physicians and seminars to practice and community nurses took place, and these had no dramatic effect on the weekly number of referrals (Figure 1). At this time the clinic was working efficiently, with two-thirds of patients being seen within two weeks of GP referral (Table I); 38% of patients seen were referred for a biopsy or other minor operation. There was substantial delay in getting these procedures completed, with over half the patients waiting over 3 months.

The launch of the public education campaign in July 1987 was followed by a massive increase in referrals, with over 120 in the first week. The referral rate was 54.8 per week in the 3 months immediately after the campaign, and in the following 3 months was still much higher than in the pre-campaign stage, namely 22.8 referrals per week. Extra clinics, up to three a week, were held to deal with this demand, but even so the delay time from GP referral to clinic appointment rose, with over half the patients waiting more than 2 weeks in the periods after the campaign. The proportion referred for minor operation or biopsy fell to 25% but there was still an increased demand. Within broad categories this did not lead to an increased delay from appointment to biopsy.

Further analysis was confined to patients referred who were residents of Nottingham Health District, to avoid problems of a changing catchment area. The proportion of total patients referred who were Nottingham residents

**Table I** Pigmented lesion clinic referrals before, and for two time periods after the public campaign, in July 1987

| | *Time period* | | |
|---|---|---|---|
| | *Before campaign 1/1/87 to 7/7/87 no. (%)* | *After 1 8/7/87 to 30/9/87 no. (%)* | *After 2 1/10/87 to 31/12/87 no. (%)* |
| Referrals, number | 272 | 658 | 296 |
| Average per week | 10.1 | 54.8 | 22.8 |
| Delay from GP referral to clinic appointment | | | |
| <2 weeks | 182 (66.9) | 162 (24.6) | 140 (47.3) |
| 2 weeks to 1 month | 70 (25.7) | 392 (59.6) | 140 (47.3) |
| >1 month | 20 (7.4) | 104 (15.8) | 16 (5.4) |
| Referral for biopsy or minor operation | 103 (37.9) | 162 (24.6) | 71 (24.0) |
| Delay from clinic appointment to biopsy | | | |
| <1 month | 22 (21.3) | 31 (19.1) | 17 (23.9) |
| 1–3 months | 25 (24.3) | 47 (29.0) | 25 (35.3) |
| >3 months | 56 (54.4) | 84 (51.9) | 29 (40.8) |
| Residents of Nottingham district (% of total) | 201 (73.9) | 520 (79.0) | 246 (83.1) |

increased after the campaign, and overall was 79%. The proportion of male patients in those referred increased after the campaign; this was due mainly to an increase in male patients aged 45 years and over.

Of patients referred, around 40% had three or more of the seven suggested danger signs and this was not altered after the campaign (Table II). In the 6 months before the public education campaign, 27% of referred patients had benign pigmented moles, and 25% seborrhoeic warts, with 31% having any of a wide range of other diagnoses. Subsequent to the campaign the proportion with benign moles was little changed, the proportion with seborrhoeic warts increased, and the proportion with miscellaneous other diagnoses decreased. Before the campaign, nine patients were seen who had melanoma (4.5% of all referrals), and in the 6 months after the campaign 14 such patients were seen (2% of all referrals). Even before the campaign, 71% of patients referred had heard of the dangers of moles, but after the campaign this was significantly increased, initially to 89%; the proportion of patients who were moderately or very worried about their lesion was not substantially changed from the pre-campaign figure of 33%.

*All dermatological referrals*

The surveys of all patients referred with pigmented lesions to dermatologists in October/November 1986, repeated in October/November 1987, shows that the increase in referrals to the new pigmented lesion clinic was in addition to rather than instead of referrals to other clinics. In October/November 1986, 169 patients with pigmented lesions were referred, of whom 159 attended for consultation. In October/November 1987, 389 patients were referred; 174 to dermatological clinics other than the PLC, and 215 to the PLC. The distribution of diagnoses in patients seen in October/November 1986 was generally similar to that of patients seen in the PLC in the pre-campaign phase, including five patients (3%) who had a cutaneous melanoma. In the patients seen in October/November 1986, the delay before the dermatology clinic appointment was substantially longer than that for the PLC, with 54% of patients waiting longer than 1 month; the referral rate for biopsy or minor operation was higher (59%), but the waiting time from appointment to biopsy was slightly shorter, with 30% waiting less than 1 month, 32% from 1 to 3 months, and 37% longer than 3 months.

*GP survey data*

Twenty-two general practitioners from five group practices recorded details of patients presenting with discrete skin lesions in the 3 weeks before the public education campaign and the 3 weeks after the campaign. Twenty-nine patients were seen in the 3 weeks before the campaign, a consultation rate of about 0.4 cases per general practitioner per week. In the week immediately after the campaign, 60 consultations were recorded, three cases per general practitioner per week, and in the following 2 weeks, 64 consultations, 1.4 consultations per general practitioner per week.

*Patients with melanoma*

The data on all patients with primary cutaneous melanoma who were residents of Nottingham Health District show an increase in frequency from 1984 to 1986, from 27 to 42 cases per year, which gives crude incidence rates per 100,000 residents of 4.4 increasing to 6.9 per year (Table III). In 1987, during which the new developments took place, 46 cases were seen, giving an incidence of 7.5, higher than previous years but not inconsistent with the previous trend. The depth distribution of tumours in patients seen after the education campaign shows a higher proportion of thin tumours than previously, but the distribution is not significantly different from that of the previous 3½ years; nor is there a difference between the depth distribution of cases diagnosed in the first and in the latter 6 months of 1987. However, because of the small numbers, effects would have to be very large to be detectable. Of the 46 patients diagnosed in 1987, 23 were referred via the PLC. Their depth distribution was similar to that of the other patients. Three of the nine PLC patients diagnosed before the campaign said they were encouraged to visit their doctor by relevant

**Table III** Primary cutaneous melanoma diagnosed in Nottingham Health District residents 1984–1987 – depth distribution[a]

| | <0.76 (%) | 0.76–1.49 (%) | 1.5–3.49 (%) | >3.5 (%) | Total | Annual incidence rate per 100,000 residents |
|---|---|---|---|---|---|---|
| 1984 | 9 (33.3) | 5 (18.5) | 8 (29.6) | 5 (18.5) | 27 | 4.4 |
| 1985 | 8 (23.5) | 11 (32.3) | 7 (20.6) | 8 (23.6) | 34 | 5.6 |
| 1986 | 19 (45.2) | 8 (19.0) | 9 (21.4) | 6 (14.3) | 42 | 6.9 |
| 1987 | 21 (45.6) | 9 (19.6) | 10 (21.7) | 6 (13.0) | 46 | 7.5 |
| 1984 to July 1987, before campaign | | | | | | |
| | 45 (36.3) | 29 (23.4) | 29 (23.4) | 21 (16.9) | 124 | |
| July to Dec. 1987, after campaign | | | | | | |
| | 12 (48.0) | 4 (16.0) | 5 (20.0) | 4 (16.0) | 25 | |

$\chi^2 = 1.37$, d.f. = 3. [a]Depths in mm.

**Table II** Pigmented lesion clinic referrals before and in two time periods after the public campaign, Nottingham residents only

| | Time period | | |
|---|---|---|---|
| | *Before campaign 1/1/87 to 7/7/87 no. (%)* | *After 1 8/7/87 to 30/9/87 no. (%)* | *After 2 1/10/87 to 31/12/87 no. (%)* |
| No. of Nottingham residents seen at clinic | 201 | 520 | 246 |
| Male referrals | 62 (30.8) | 203 (39.0) | 81 (32.9) |
| Appropriate referrals[a] (≥3 danger signs) | 75 (39.7) | 186 (39.2) | 86 (39.1) |
| Diagnosis | | | |
| Benign pigmented moles | 54 (26.9) | 137 (26.3) | 63 (25.6) |
| Seborrhoeic warts | 51 (25.4) | 174 (33.5) | 68 (27.6) |
| Melanoma (NM + SSM) | 9 (4.5) | 9 (1.7) | 5 (2.0) |
| Other | 62 (30.8) | 106 (20.4) | 65 (26.7) |
| Not yet established | 25 (12.4) | 94 (18.1) | 45 (18.3) |
| Heard of danger?[b] | 136 (70.8) | 447 (88.9) | 188 (81.3) |
| Very or moderately worried[c] | 64 (33.3) | 156 (30.5) | 80 (35.4) |

[a]Before campaign 189 cases suitable for analysis (94%); after campaign (1) 475 (91.2%), (2) 220 (89.4%).
[b]Before campaign 192 responders (95.5%); after campaign (1) 503 (96.5%); after campaign (2) 231 (93.9%).
[c]Before campaign 192 responders (95.5%); after campaign (1) 501 (96.3%); after campaign (2) 226 (91.9%).

publicity, and this was reported by six of 14 patients diagnosed after the campaign. The great majority of patients presenting to the PLC with melanomas, both before and after the campaign, had been aware of their skin lesions for a considerable length of time without taking action; 11 (48%) for 1 year or more; nine (39%) for between 3 months and 1 year. For only three patients (13%) is it possible that the campaign influenced the first recognition of the abnormality.

### Discussion

The clearest effect of this public education campaign about melanoma has been the dramatic increase in referrals to the PLC (Figure 1). The effect of this locally targeted campaign was much more dramatic than the effect of the publication of the Royal College of Physicians' report, which received considerable national press coverage and some local media coverage. Although waiting times for consultation at the PLC increased with this great increase in referrals, over 80% of patients referred were seen within 1 month, a considerable improvement in the time taken for consultations for dermatological patients with similar lesions before the inception of the PLC. Dealing with these increased referrals put considerable strain on nursing, medical and clerical resources. Without the PLC and the full co-operation of the staff involved, this campaign would have led to a chaotic situation within the dermatological service. This justifies the decision of the Cancer Research Campaign's Steering Group not to run new educational campaigns until improvements in services have been made, and this lesson will be relevant for future campaigns.

The campaign resulted in some shift of diagnosis and management patterns, with a higher proportion of patients with seborrhoeic warts being seen, and a fall in the proportion of patients biopsied from 38 to 25%. However, the number of patients requiring minor surgery increased from 3.8 per week before the campaign to 13.5 per week in the 3 months immediately following the campaign, and 5.5 per week in the subsequent 3 months, creating a substantial pressure on minor operating facilities, leading to longer delays in general for dermatological procedures than before the inception of the PLC. Throughout this period, lesions strongly suspected of being a melanoma were removed within 1 week.

The proportion of patients who were seen at the PLC who had melanoma was 4.5% before the campaign, and 2% subsequently. Efforts to make referral more specific for patients who do have early melanoma would be useful, particularly if public education campaigns are to be conducted. Of the 23 patients seen with melanoma at the PLC, 21 fulfilled Mackie's criterion of having at least three out of seven warning signs present (Mackie, 1986). Of the 861 other patients seen at the clinics, 326 (38%) had three or more warning criteria. Thus in patients referred who fulfilled this criterion, 6% (21/347) had melanoma, compared to 0.4% (2/537) of patients not meeting the criterion; 99.6% of patients referred without at least three signs did not have melanoma. Although based on inadequate numbers for formal evaluation, this evidence suggests that this simple checklist and decision criterion is useful, and could be given more emphasis in future campaigns. However, there is room for improvement, and a refinement of the checklist to achieve greater specificity should be possible, with increasing knowledge about the early signs and symptoms of melanoma.

In contrast to the dramatic, and potentially disruptive, effect on dermatological referrals, the effect on visits to general practitioners was minor, an increase from approximately one patient every 2 weeks to three per week immediately following the campaign; this level of increase should be easy for general practitioners to deal with, if they have been informed of it in advance and if the facilities for referral have been provided and adequately publicised.

The campaign appeared to result in a substantial increase in the proportion of patients referred to the PLC who had recently heard of the danger of moles, although this proportion was quite high (71%) before the campaign; we cannot tell if this is a reflection of the effect of the campaign on the whole population, or whether these patients might have been informed of dangers by the GPs or other primary care staff who in turn had been alerted by the campaign. The proportion of patients who said that they were moderately or very worried did not change substantially, and although this means that a greater number of people who regarded themselves as worried were referred to clinics, it suggests that the deleterious effects of the campaign, in terms of producing unwelcome worry, are likely to be minor.

The current data are insufficient to assess the impact of the campaign on the depth distribution of melanomas, and demonstrates some of the difficulties in this assessment. From 1984 to 1986 there has been a noticeable increase in the incidence of melanoma in this district, and the data show a trend to diagnosis of thinner tumours, although this is not statistically significant. The data for the whole of 1987 show a further increase in incidence, and a similar depth distribution as in previous years. In the 3½ years before the campaign, 58% of tumours were less than 1.5 mm thick; this proportion is similar to that achieved in Glasgow and in Trentino, Italy, *after* public education campaigns. Although there had been no specific local educational efforts in regard to melanoma before 1987, the publicity given to previous campaigns in Glasgow and elsewhere may have had some beneficial effect in Nottingham (Mackie & Doherty, 1988). These trends may also result from increases in public awareness which cannot be specifically traced to any one effort.

Evaluation of the effects of educational campaigns on the depth distribution of melanoma, and ultimately on the death rate from melanoma, requires studies covering much larger populations and much longer time periods. The optimal scientific design of a randomised trial has not been developed anywhere; as a development from the current work a national evaluation project comparing a number of areas including Nottingham with control areas is being undertaken. Even this evaluation may be difficult to achieve because the effect of specific campaigns is likely to extend to non-targeted areas.

In conclusion, the public education campaign mounted in Nottingham has had dramatic effects on the number of patients referred for assessment of pigmented lesions, and this would have caused severe problems if adequate resources had not been made available to deal with it. While a substantial proportion of patients presenting with melanoma state that their presentation was precipitated by the campaign, the data are insufficient as yet to show an improvement in depth distribution. Most patients with melanoma who presented as a result of the campaign had been conscious of having lesions for a considerable time, so that the campaign appears to precipitate action in regard to lesions already noticed, rather than to make people notice abnormalities they have previously not recognised. We feel that the continuing increase in mortality from melanoma justifies the use of such education campaigns, while the current work emphasises the service implications, and suggests that more specific targeting, particularly on guidelines for referral by general practitioners, might be very beneficial. Evaluation of the effects of such campaigns, which requires large scale and long-term studies, is essential as their beneficial effects cannot be taken for granted.

We thank all Nottingham dermatologists and pathologists for their co-operation, and general practitioners from the five participating group practices. Thanks also to Jean Jones for organising the pigmented lesion clinic, Alison Langham and Myra Galt for help with data collection and coding, and Joyce Gilbert for preparation of the manuscript. The project was supported by a grant from the Cancer Research Campaign.

### References

BRESLOW, H. (1970). Thickness, cross-sectional area and depth of invasion in the prognosis of cutaneous melanoma. *Ann. Surg.*, **172**, 902.

CRISTOFOLINI, M., ZUMIANI, G., BOI, S. & PISCIOLI, F. (1986). Community detection of early melanoma. *Lancet*, **i**, 156.

DOHERTY, V.R. & MACKIE, R.M. (1986). Reasons for poor prognosis in British patients with cutaneous malignant melanoma. *Br. Med. J.*, **292**, 987.

GALLAGHER, R.P., ELWOOD, J.M. & HILL, G.B. (1986). Risk factors for cutaneous malignant melanoma: the Western Canada Melanoma Study. *Rec. Results Cancer Res.*, **102**, 38.

GORDON, L.G. & LOWRY, W.S. (1986). Missed malignant melanomas. *Br. Med. J.*, **292**, 1524 (letter).

HOLMAN, C.D.J., ARMSTRONG, B.K., HEENAN, P.J. and 11 others (1986). The causes of malignant melanoma: results from the Western Australian Lions Melanoma Research Project. *Rec. Results Cancer Res.*, **102**, 18.

MACKIE, R.M. (1986). *An Illustrated Guide to the Recognition of Early Malignant Melanoma.* University of Glasgow.

MACKIE, R.M., ELWOOD, J.M. & HAWK, J.L.M. (1987). Links between exposure to ultraviolet radiation and skin cancer. A report of the Royal College of Physicians, London.

MACKIE, R.M. & DOHERTY, V.R. (1988). Educational activities aimed at earlier detection and treatment of malignant melanoma in a moderate risk area. In *Melanoma and Naevi*, Elwood, J.M. (ed) p. 140. Karger: Basel.

OSMOND, C., GARDNER, M.J., ACHESON, E.D. & ADELSTEIN, A.M. (1983). *Trends in Cancer Mortality Analyses by Period of Birth and Death 1951–80.* HMSO: London.

SCHNEIDER, J.S., SAGABIEL, R.W., MOORE, D.H. & LAWTON, G.M. (1987). Melanoma surveillance and earlier diagnosis. *Lancet*, **ii**, 1435.

SMITH, A. (1979). The Queensland Melanoma Project – an exercise in health education. *Br. Med J.*, **i**, 253.

SOUTHAMPTON MELANOMA GROUP (1986). Effect of rapid referral on thickness of melanomas. *Br. Med. J.*, **293**, 790.

TEMOSHOK, L., CLEMENTE, R.J., SWEET, D.M., BLOIS, M.S. & SAGABIEL, R.W. (1984). Factors related to patient delay in seeking medical attention for cutaneous malignant melanoma. *Cancer*, **54**, 3048.